# Distributions of Extracellular Peptidases Across Prokaryotic Genomes Reflect Phylogeny and Habitat

**DOI:** 10.3389/fmicb.2019.00413

**Published:** 2019-03-05

**Authors:** Trang T. H. Nguyen, David D. Myrold, Ryan S. Mueller

**Affiliations:** ^1^Department of Crop and Soil Science, Oregon State University, Corvallis, OR, United States; ^2^Department of Microbiology, Oregon State University, Corvallis, OR, United States

**Keywords:** protease, peptidase, protein, secreted enzymes, extracellular enzymes, phylogeny

## Abstract

Proteinaceous compounds are abundant forms of organic nitrogen in soil and aquatic ecosystems, and the rate of protein depolymerization, which is accomplished by a diverse range of microbial secreted peptidases, often limits nitrogen turnover in the environment. To determine if the distribution of secreted peptidases reflects the ecological and evolutionary histories of different taxa, we analyzed their distribution across prokaryotic lineages. Peptidase gene sequences of 147 archaeal and 2,191 bacterial genomes from the MEROPS database were screened for secretion signals, resulting in 55,072 secreted peptidases belonging to 148 peptidase families. These data, along with their corresponding 16S rRNA sequences, were used in our analysis. Overall, *Bacteria* had a much wider collection of secreted peptidases, higher average numbers of secreted peptidases per genome, and more unique peptidase families than *Archaea*. We found that the distribution of secreted peptidases corresponded to phylogenetic relationships among *Bacteria* and *Archaea* and often segregated according to microbial lifestyles, suggesting that the secreted peptidase complements of microbial taxa are optimized for the environmental microhabitats they occupy. Our analyses provide the groundwork for examining the specific functional role of families of secreted peptidases in relationship to the organisms and the corresponding environments in which they function.

## Introduction

Peptidases catalyze the cleavage of the peptide bonds between amino acid residues of proteins and are produced by all forms of life (Rao et al., 1998). These proteolytic enzymes are highly diverse in structure, perform multiple biological functions, and can be found in the cytoplasm within cells, tethered to the cell surface, or secreted into the environment. Secretion of extracellular peptidases represents a significant investment of metabolic energy, carbon, and nitrogen by microbial cells enabling the acquisition of carbon or nitrogen from the environment (Chróst, 1991; Kumar and Takagi, 1999; Geisseler and Horwath, 2008; Allison et al., 2010; Landi et al., 2011).

Proteinaceous material is the most abundant form of soil organic nitrogen. Protein degradation into oligopeptides and amino acids, which can be directly and rapidly metabolized by microorganisms for nutrients and energy, is a critical strategy used by microorganisms to gain bioavailable nitrogen under nitrogen-limited conditions, especially in boreal and temperate forest soils (Schimel and Bennett, 2004; Geisseler et al., 2010). Some peptidases are secreted constitutively into the environment at low concentrations by microorganisms to initiate the degradation of proteins, although microorganisms can also regulate peptidase production and secretion based on their demands for carbon and nitrogen (Geisseler and Horwath, 2008).

In aquatic ecosystems, proteins and peptides contribute significantly to dissolved organic matter, accounting for 5–20% of dissolved organic nitrogen and 3–4% of dissolved organic carbon (Nagata et al., 1998; Pantoja and Lee, 1999). As in terrestrial ecosystems, microbial utilization of organic nitrogen in aquatic systems is regulated by the hydrolysis of these protein polymers (Chróst, 1991). Due to the more dilute nature of aquatic environments, peptidases bound to microbial cells and sequestered in microbial biofilms are thought to be primarily responsible for the degradation of proteins, and allow microbes to readily take up the decomposition products for further metabolism (Chróst, 1991; Hoppe, 1991; Nagata et al., 1998; Nunn et al., 2003), however, free proteolytic enzymes also contribute to the available nitrogen pool (Chróst, 1991; Obayashi and Suzuki, 2008).

In animal-associated environments protein degradation can be associated with pathogenicity and host disease (Gibson and Macfarlane, 1988b; Richardson et al., 2013), in addition to having a role in direct nutrient acquisition. In some gut environments, microbial peptidases have been found to be constitutively produced and partially bound to the cell surface (Macfarlane et al., 1986; Gibson and Macfarlane, 1988a,b). The regulated secretion of some extracellular peptidases is proposed to help pathogenic microorganisms competitively colonize and invade host cells and tissues by degrading host proteins, such as mucins, collagens, and other extracellular-matrix components (Gibson and Macfarlane, 1988a; Loesche, 1988; Nakjang et al., 2012; Duarte et al., 2016).

Peptidases are universal across all organisms and are considered to have developed early during biological evolution (Rao et al., 1998). Subsequent diversification has led to the development of several peptidase super-families (asparagine, aspartic, cysteine, glutamic, metallo-, serine, and threonine peptidases) that are grouped based on their mechanism of catalysis (Hartley, 1960; Häse and Finkelstein, 1993; Rawlings and Barrett, 1993; Rao et al., 1998; Mooshammer et al., 2014; Rawlings, 2016; Rawlings et al., 2018). Different classes of peptidases are associated with specific biological pathways, substrates, and catalytic reactions (Rawlings and Barrett, 1993; Rao et al., 1998; Page and Cera, 2008). Peptidase families have been shown to be distributed unevenly among microbial groups (Page and Cera, 2008), leading to broad generalizations about associations of peptidases – both intracellular and extracellular – with different microbial groups. Aspartic peptidases are mostly encoded by *Fungi*, metallopeptidases are common in *Bacteria*, and cysteine and serine peptidases appear to be universal across microorganisms (Caldwell, 2005). *Bacteria* have consistently been found to be the dominant contributor to protease activity in soils and seawater based on studies using protease activity assays, pure culture protease expression, and approaches targeting peptidase genes (Watanabe and Hayano, 1993, 1994, 1996; Katsuji et al., 1994; Nagata et al., 1998; Watanabe et al., 2003; Sakurai et al., 2007; Obayashi and Suzuki, 2008). However, it is unclear how varying peptidase complements across genomes may impact variations in overall activity when considered at a community level or inferred by metagenomics studies. By developing a better understanding of factors influencing the abundance, diversity, and distribution of extracellular peptidase genes across all curated prokaryotic taxa (Burns et al., 2013; Arnosti, 2015), insights into the relationship between microbial community composition and protein degradation capabilities across environments can be gained.

Our goal was to analyze the diversity of secreted peptidases and their distributions across prokaryotic microorganisms by using annotated peptidase sequences collated in the MEROPS database (Rawlings, 2016; Rawlings et al., 2018). We expected to find secreted peptidases from different proteolytic super-families distributed widely across the prokaryotic tree of life. From this peptidase distribution pattern, we also sought to find evidence of whether each peptidase family is evolutionarily conserved among phylogenetically-related taxa. Because the catalytic efficiencies of secreted peptidases from different super-families are known to be affected by environmental conditions, we expected the distribution of peptidases to also vary as a function of the ecological microhabitats occupied by different microbial taxa. More broadly, the findings from these analyses might provide fundamental insights into the complement of secreted peptidases in microorganisms within different environments, which could be further validated using assays of peptidase gene expression and proteolytic activity.

## Materials and Methods

### Collection of Secreted Archaeal and Bacterial Peptidases and Signal Sequence Identification

Annotated peptidase sequences of 147 archaeal and 2 191 bacterial species were extracted from the MEROPS MySQL database release 11.0 (Rawlings, 2016)^1^. Only completely annotated genomes with available 16S rRNA information existing in SILVA database release 128 were considered. Firstly, data pertaining to the organism name, taxonomy, and genome completeness was extracted from the “organism” and “classification” tables of the MySQL database (i.e., merops_taxonomy_id, taxonomy_id, and complete_genome values). The taxonomy_id values were used to query for matching 16S rRNA sequences from the SILVA database. Unique taxonomic IDs present in both databases and encoding at least one secreted peptidase were used as the primary genomes of interest for this study.

The MEROPS database classifies peptidases into seven super-families based on the catalytic residue serving at the active site of the enzyme (Hartley, 1960; Rawlings and Barrett, 1993), and further divides these super-families into 255 proteolytic families based on similarities in amino acid sequences (Rawlings, 2016). The merops_taxonomy_id was used as a search query against the “features” and “sequence” tables of the MEROPS MySQL database to obtain all information pertaining to annotated peptidase sequences encoded within each genome of interest. Exported information included the peptidase DNA sequence, the sequence_id, and the peptidase super-family and family classification.

All downloaded peptidase sequences were analyzed with SignalP 4.1 to identify genes encoding putative signal sequence motifs as defined for Gram-positive and Gram-negative bacteria (Mori and Ito, 2001; Petersen et al., 2011), yielding 55,072 secreted peptidases classified to 148 families. To validate the signal peptide prediction using SignalP, we analyzed these 55,072 sequences with Phobius, a combined transmembrane topology and signal peptide predictor, which has a reported higher sensitivity in discriminating between transmembrane domains and signal peptides (Käll et al., 2004, 2007). More than 98% of the sequences identified by SignalP were also identified as having signal peptides by Phobius, 0.6% were identified as not having either signal peptides or transmembrane domains, and 1.3% were identified as transmembrane proteins with any signal peptides. Taking into account the imperfection that exists in all signal prediction models, we concluded that using SignalP was a valid method to identify prokaryotic secreted peptidases in our study. Peptidase sequences of *Firmicutes*, *Actinobacteria*, *Deinococcus-Thermus*, and *Archaea* were screened using a Gram-positive model; the remaining bacterial peptidase sequences were screened using a Gram-negative model. The Welch two-sample *t*-test, or unequal variances *t*-test, was used to evaluate significant difference between the means of the total secreted peptidases encoded within archaeal and bacterial genomes. One-way analysis of variance with the Tukey’s HSD multiple-range test was used to determine the statistical differences between counts of total secreted peptidases among microbial phyla. Statistical analyses were performed in the “R” programming environment (R Core Team, 2016).

### Comparison of Genomic Complements of Secreted Peptidases

The secreted peptidase complements of all taxa were summarized in a matrices containing the gene copy number counts of secreted peptidases assigned to either family or superfamily classifications (rows) across all analyzed genomes (columns). Bray-Curtis dissimilarity indices between the secreted peptidase complements of genomes were calculated from these matrices and used to generate a secreted peptidase distance matrix, or functional distance matrix, using the “Vegan” package in “R” (Oksanen et al., 2018). Principal coordinate analyses (PCoA) was used to explore the data and Permutational Multivariate Analyses of Variance (PERMANOVA) was used to determine the statistical differences of the peptidase complements of archaeal and bacterial genomes at different taxonomic levels. A bipartite association network of shared and unique peptidase families was generated using Cytoscape release 3.4.0^2^ (Shannon et al., 2003).

### Phylogenetic Analysis

The 16S rRNA sequences of the selected archaeal and bacterial genomes were extracted from the SILVA database release 128^3^ (Quast et al., 2013) and aligned using the NAST aligner (DeSantis et al., 2006). A 16S rRNA neighbor-joining phylogenetic tree was built from alignments using PHYLIP (Felsenstein, 1989). A phylogenetic distance matrix was also constructed using the F84 model of DNADIST (DeSantis et al., 2006). The phylogenetic tree and distributions of secreted peptidase families across the tree were visualized using iTOL (Letunic and Bork, 2016).

### Distance Matrices Comparisons

Correlations between the phylogenetic distance matrix and the secreted peptidases distance matrix, or functional distance matrix, were evaluated using the Mantel test of “APE” (Analysis of Phylogenetics and Evolution package) in “R” (Paradis et al., 2004) based on Pearson’s product-moment correlation. Mantel correlograms that report the correlation between phylogenetic and functional distances at defined phylogenetic distance classes for *Archaea* and *Bacteria* were calculated using the “Vegan” package in “R.”

### Phylogenetic Conservation and Clustering

Phylogenetic signal strengths (D) contributing to the observed distribution patterns for each peptidase super-family and family were calculated from their binary presence/absence in genomes of all considered taxa (Fritz and Purvis, 2010) using the “CAPER” package (Comparative Analyses of Phylogenetics and Evolution) in “R” (Orme et al., 2018). Secreted peptidases are considered phylogenetically conserved when they are shared among the majority of members of deeply branched clades, conforming to a Brownian motion evolutionary model (D ∼ 0), with a relatively constant gain/retention of traits across taxonomic levels. A strongly clumped distribution (D < 0) suggests recent innovation or potential gain via horizontal gene transfer within a clade or subset therein. Peptidases are considered randomly distributed (D ≥ 1) when their presence/absence is not driven by shared traits (e.g., microhabitat, physiology) of closely related species (Berlemont and Martiny, 2013; Martiny et al., 2013; Zimmerman et al., 2013).

### Phylogenetic Conservation of Secreted Peptidases in Association With Microbial Habitats

To understand the association between the distribution of secreted peptidases and ecological microhabitats, we examined taxonomic subsets of microorganisms, including genomes of 147 *Archaea*, 275 *Actinobacteria*, and 182 *Bacteroidetes* species. Habitat preferences for archaeal phyla and proposed optimal growth conditions (e.g., pH, temperature, and salt concentration ranges) and for bacteria were downloaded from the JGI GOLD database (Mukherjee et al., 2017). Habitat preferences were visualized on phylogenetic trees together with secreted peptidase count data using iTOL. For *Actinobacteria* and *Bacteroidetes* datasets, the Welch two-sample *t*-test was used to determine statistical differences between the mean gene copy number of each secreted peptidase super-family between microbial groups originating from different ecological habitats (e.g., soil and aquatic habitat vs. animal-associated habitat). PCoA was used to visualize the data in multidimensional space and PERMANOVA was used to determine the statistical differences of the peptidase complements of microbial groups originating from different ecological habitats. Vectors for peptidase families capturing a significant amount of variation in the total dataset were derived from Pearson correlations with the first two PCoA axes.

## Results

### Distribution of Secreted Peptidases Across Prokaryotic Kingdoms

**FIGURE 1:**
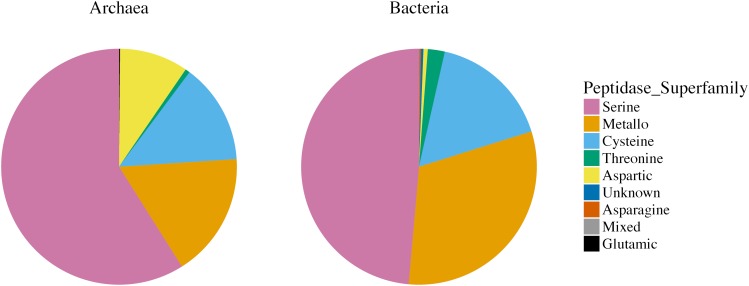
Relative abundance of secreted peptidase super-families in 147 archaeal and 2,191 bacterial genomes (asparagine, aspartic, cysteine, glutamic, metallo-, mixed, serine, threonine, and unknown peptidase super-families). Different colors represent different peptidase super-families.

**FIGURE 2:**
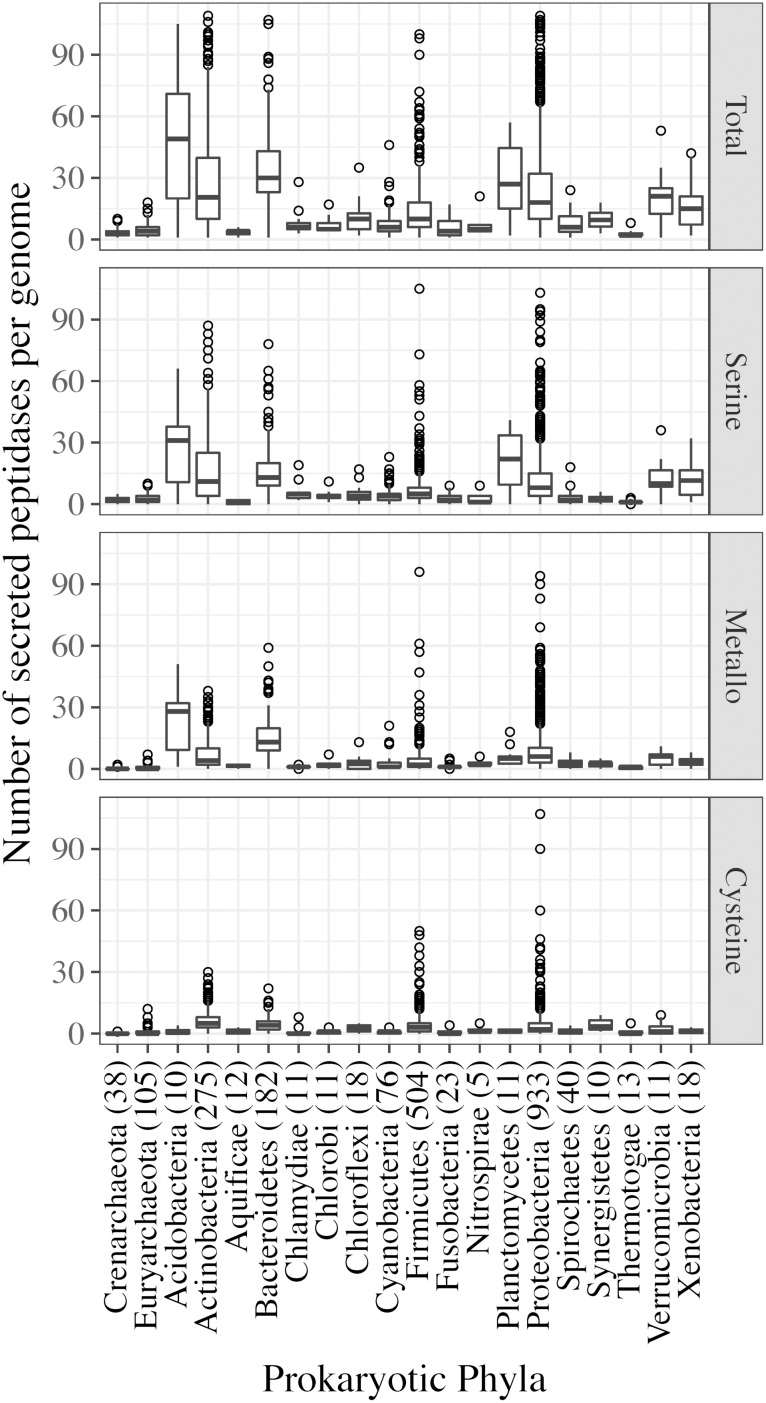
Secreted peptidase gene content (per genome) of archaeal and bacterial phyla. Secreted peptidases were grouped into super-families: Total secreted peptidases (including genes from all peptidase super-families); serine, metallo-, cysteine peptidases. The number of analyzed genomes from each prokaryotic phylum is presented next to the phylum names.

When normalized to genome size, *Bacteria* had significantly more secreted peptidase coding genes per Mb than *Archaea* (5.84 vs. 1.71, *p* < 0.001) (Supplementary Figure S1). In both kingdoms, serine, metallo-, and cysteine super-families contributed more than 80% of the secreted peptidase genes (Figure 1). The numbers of peptidase genes per genome belonging to these abundant super-families were also significantly lower in archaeal than in bacterial phyla, agreeing with the general trends of kingdom-level peptidase super-family repertoires (Figure 2). Conversely, significant biases were observed in some of the less common peptidase super-families: aspartic peptidases were more common in *Archaea* than *Bacteria* (9.4% vs. 0.6%), whereas threonine peptidases were more commonly found in *Bacteria* than *Archaea* (2.3% vs. 0.6%) (Figure 1 and Supplementary Figure S2). Asparagine, glutamic, mixed, and unknown peptidase super-families were rare (Figure 1).

**FIGURE 3:**
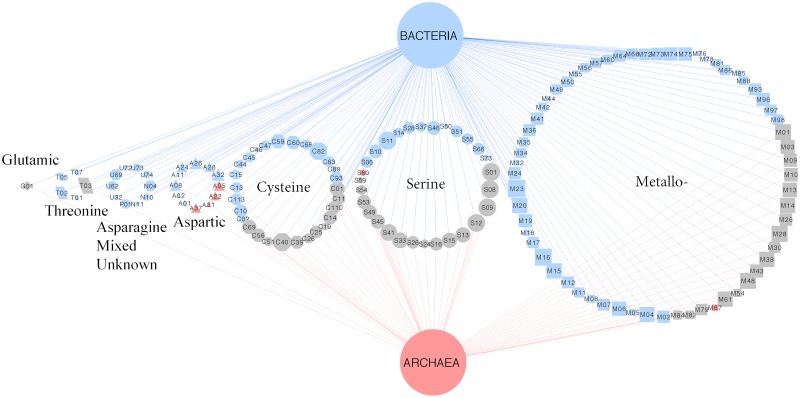
Bipartite association network of shared peptidase families between *Archaea* and *Bacteria*. Node sizes indicate the relative abundance of the secreted peptidases. Node shapes represent different peptidase families: triangle, aspartic; octagon, cysteine; diamond, glutamic; rectangle, metallo-; pentagon, serine; parallelogram, threonine; hexagon, asparagine, mixed, and unknown. Node colors are coded by unique or shared peptidase families between microbial kingdoms (blue, *Bacteria*; red, *Archaea*; gray, shared between *Bacteria* and *Archaea*). Edges denote associations between microbial kingdoms and peptidase families. Edge colors are coded by microbial kingdoms.

Observing the distribution of peptidase families, as opposed to super-families, offered finer-scale insights into the differential sets of secreted peptidases encoded by *Archaea* and *Bacteria*. Most of peptidase families encoded by *Archaea* were also common to *Bacteria*: 47 peptidase families of the serine, metallo-, cysteine, threonine, and glutamic super-families were shared between the two kingdoms, and contributed to more than one-third of the total peptidase families present in the dataset (Figure 3). Only six peptidase families were unique to *Archaea*, four belonging to the aspartic super-family, whereas 95 peptidase families were unique to members of the bacterial kingdom (Figure 3).

**FIGURE 4:**
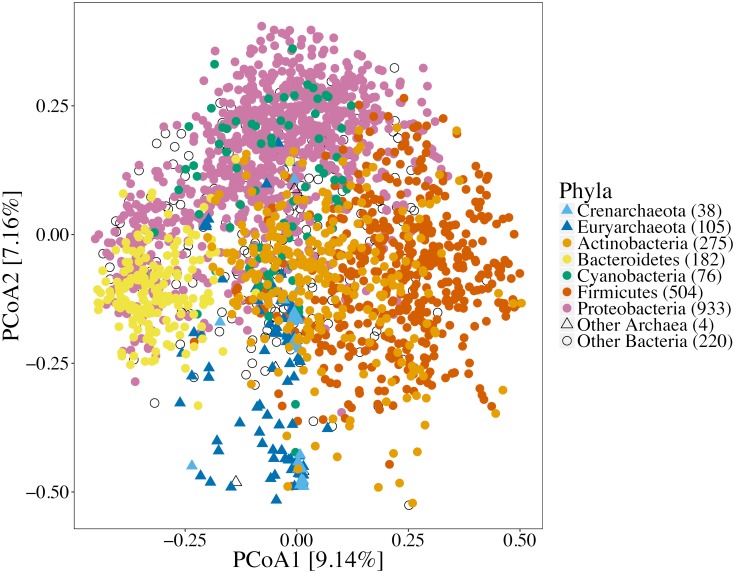
Principal coordinate analysis of secreted peptidase families based on Bray-Curtis dissimilarity of proportions of secreted peptidase families encoded in each genome. Symbol shapes are coded by microbial kingdoms; symbol colors represent some abundant bacterial and archaeal phyla. Significant differences of the secreted peptidase profiles were observed between archaeal and bacterial species (*p* < 0.001, F-statistic = 92.9, PERMANOVA) and among different bacterial phyla (*p* < 0.001, F-statistic = 19.0, PERMANOVA).

Principal coordinate analysis clustered phylogenetically distinct sets of microorganisms separate from each other based on the secreted peptidase families they encode (Figure 4). Although each PCoA axis explained a low level of data variance, PERMANOVA tests indicated significant differences between different sets of microorganisms. For example, a strong and significant difference of the secreted peptidase profiles was observed between *Archaea* and *Bacteria* (*p* < 0.001) and between bacterial phyla (*p* < 0.001).

Distributions of peptidase families and corresponding super-families across microbial taxa were compared to the phylogenetic relationships among analyzed genomes using presence/absence profiles of peptidases in comparison to a 16S rRNA phylogenetic tree. The distributions of secreted peptidases were found to be significantly correlated with the 16S rRNA phylogeny within each kingdom (*Archaea* r_Mantel_ = 0.303, *p* < 0.001, *Bacteria* r_Mantel_ = 0.334, *p* < 0.001), indicating an evolutionary relationship in which subsets of phylogenetically related organisms in each prokaryotic kingdom shared similar types of secreted peptidases. Mantel correlograms showed that conservation was strongest and most significant between more closely related taxa for both *Archaea* and *Bacteria* (Supplementary Figure S3). *Archaea* demonstrated a weakly significant relationship at all taxonomic levels examined, whereas relationships within *Bacteria* were weakly significant only between taxa that share ≥90% 16S rRNA gene sequence identity, beyond which pairs of taxa share little to no functional similarity in the secreted peptidases they encode (Supplementary Figure S3).

Distributions of individual secreted peptidase families were also evaluated for their phylogenetic dispersion (D). Most of peptidase families (71%) encoded in bacterial genomes showed evidence of non-random phylogenetic clustering (Supplementary Figure S4 and Supplementary Table S2). Peptidase families with negative values (D < 0) represented those with the strongest clustering patterns across the phylogenetic tree. For example, M73 and M84 are endopeptidases that are predominantly restricted to *Bacillus* sp. and M07 is an endopeptidase found mainly in *Actinobacteria* species (Supplementary Figure S5). Conversely, 77% of peptidase families found within archaeal genomes exhibited random distribution patterns, devoid of phylogenetic signals (Supplementary Figure S4 and Supplementary Table S3).

### Distribution of Secreted Peptidases Within Prokaryotic Kingdoms

**FIGURE 5:**
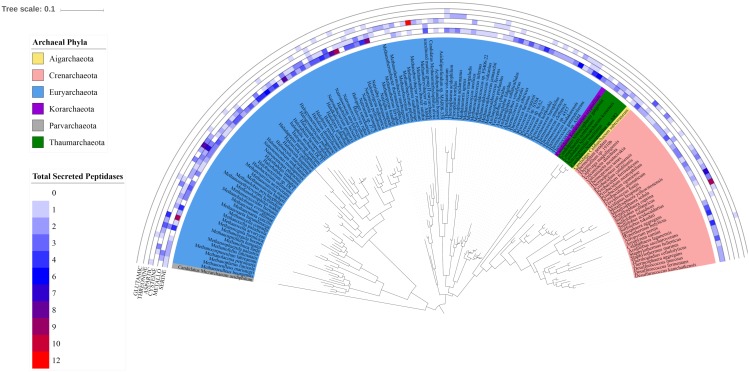
Distribution of secreted peptidase super-families across the archaeal 16S rRNA phylogenetic tree. Outer tracks show the copy number of genes from each secreted peptidase super-family in each genome. Inner track color corresponds to the phylum-level classification of each taxon considered.

There was no significant difference between the total number of secreted peptidase genes encoded in known *Crenarchaeal* and *Euryarchaeota* genomes (*p* = 0.089), however, the overall composition did vary significantly between these phyla. For example, the overabundance of aspartic peptidases observed at the kingdom-level could be primarily attributed to taxa belonging to *Crenarchaeaota*, as only 15 of 105 of *Euryarchaeota* genomes encoded these genes (Figure 5 and Supplementary Figure S2). Intriguingly, 40% of the *Euryarchaeota* genomes encoding aspartic peptidases were classified as acidophiles, whereas only 8% of the entire *Euryarchaeota* dataset fell into this environmental classification. Thus, a distinct enrichment was observed for the presence of aspartic peptidases in acidophilic *Euryarchaeota* genomes.

**FIGURE 6:**
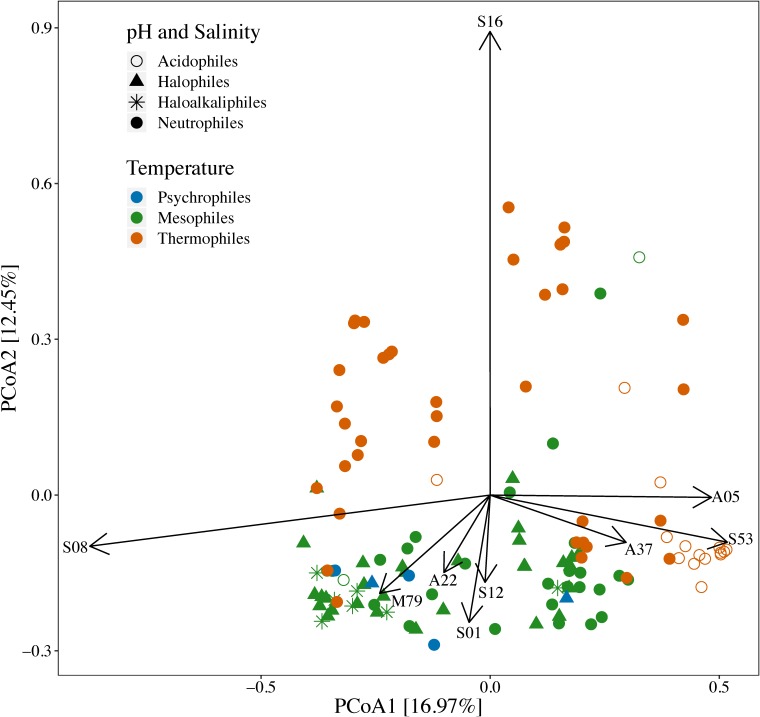
Principal coordinate analysis of prokaryotic genomes based on Bray-Curtis dissimilarities of proportions of secreted peptidase families encoded in archaeal genomes. Symbol shapes and colors are coded by reported optimal growth conditions (pH, temperature, and salt concentration). Vectors lengths are scaled relative to the correlation of individual peptidase families with the two axes shown (Pearson’s correlation). The composition of secreted peptidase genes of *Archaea* varied significantly based on their optimal growth conditions (*p* < 0.001, F-statistic = 6.11, PERMANOVA).

This link between environment and peptidase content of archaeal genomes was further explored with PCoA of peptidase profile data. The composition of secreted peptidase genes of *Archaea* varied significantly based on optimal growth conditions (pH, temperature, and salinity) (*p* < 0.001). Halophilic and haloalkilophilic archaeal clusters were most strongly correlated with the distribution of the serine S01, S08, S12, aspartic A22, and metallo M79 peptidase families (Figure 6). Thermophilic and thermophilic/acidophilic archaea also separated from each other and from the rest of archaea (Figure 6). Serine S16 peptidase family was associated with the thermophilic archaea. Following the general trend proposed above, aspartic A05 and A37 families were associated with acidophilic archaea (Figure 6). In addition, the presence of serine S53 family peptidases was strongly correlated with an acidophilic lifestyle.

**FIGURE 7:**
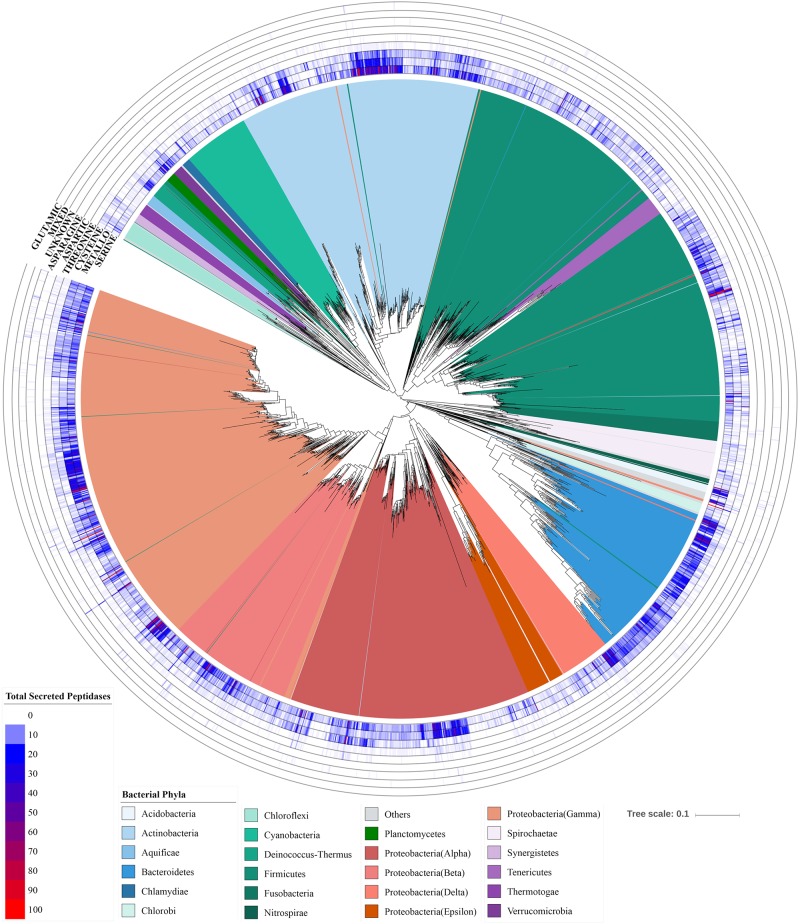
Distribution of secreted peptidases super-families across the bacterial phylogenetic tree. Outer tracks show the copy numbers of genes from each secreted peptidase super-family in each genome. Inner track color corresponds to the phylum-level classification of each taxon considered.

Secreted peptidase profiles of bacterial genomes varied substantially across taxa (Figure 7), with significant differences between phyla in total peptidase counts (Supplementary Table S1) and in composition (Figure 4). Significant differences were observed between Gram-positive and Gram-negative bacteria based on the relative abundance of their secreted peptidase families (*p* < 0.001) (Supplementary Figure S6). At the phylum level, *Acidobacteria*, *Actinobacteria*, *Bacteroidetes*, *Planctomycetes*, and *Proteobacteria* were enriched with secreted peptidases, whereas the deep-branching *Aquificae* and *Thermotogae* taxa encoded fewer secreted peptidases (Figure 2 and Supplementary Table S1). *Acidobacteria* encoded the highest mean number of secreted peptidases per genome, with a large relative increase in the number of secreted metallopeptidases and a concomitant decrease in the number of cysteine peptidases compared to other bacterial phyla (Figure 2).

**FIGURE 8:**
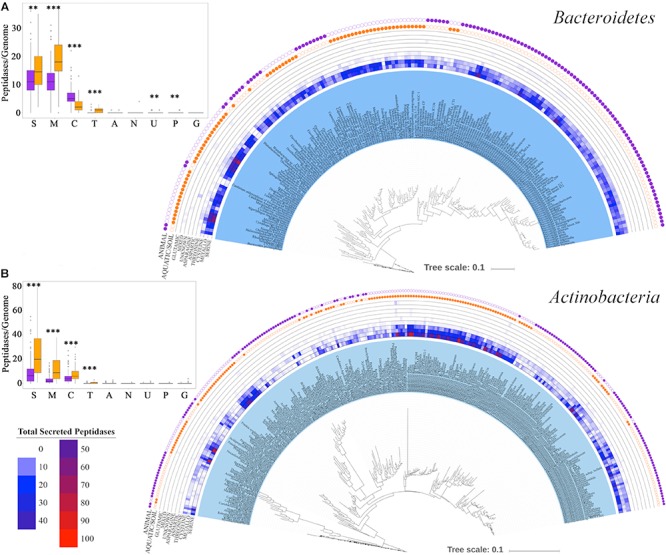
Distribution of strain-specific secreted peptidases across **(A)** *Bacteroidetes* and **(B)** *Actinobacteria* taxa. The outer two tracks represent the habitat each taxon is associated with (orange for aquatic/soil environment, purple for animal environment). The middle tracks show the copy number of genes from each secreted peptidase super-family in each genome. The figure on the top left corner represent the average number of each peptidase super-family (S for serine, M for metallo-, C for cysteine, T for threonine, A for aspartic, N for asparagine, U for unknown, P for mixed and G for glutamic) per genome; purple box plots report mean and standard deviation of the peptidase content of genomes that are commonly found in animal-associated environments, and orange box plots report mean and standard deviation of the peptidase content of genomes from soil/aquatic environments, ^∗∗∗^*p* = 0–0.001 and ^∗∗^*p* = 0.001–0.01.

Genomes of the *Bacteroidetes* and *Actinobacteria* phyla encoded high numbers of secreted peptidases and exhibited strong within-phylum distribution patterns related to finer-scale relationships (Figure 7). For the *Bacteroidetes*, serine and metallopeptidases were dominant and well-conserved in presence and copy number across all species (Figure 8A). By contrast, cysteine peptidases seemed to be more abundant (i.e., higher copy number per genome) in more recently evolved families of *Bacteroidetes*, such as *Porphyromonadaceae*, *Bacteroidaceae*, and *Prevotellaceae*, and threonine peptidases were more commonly found in deeper-branching lineages of *Bacteroidetes*, including *Cytophagaceae*, *Sphingobacteriaceae*, and *Flavobacteriaceae* (Figure 8A). The latter group of *Bacteroidetes* also had a significantly higher numbers of secreted peptidases compared to the more recently evolved group (*p* < 0.001). Principal coordinate analysis of *Bacteroidetes* showed a significant separation among *Bacteroidetes* families based on the relative abundances of secreted peptidases (*p* < 0.001), which was strongly correlated with the environment associated with each species (*p* < 0.001) (Supplementary Figure S7). All species of *Porphyromonadaceae*, *Bacteroidaceae*, and *Prevotellaceae*, which encoded fewer secreted peptidases overall but a higher proportion of cysteine peptidases, were associated with an animal environment, whereas 74% of the *Cytophagaceae*, *Sphingobacteriaceae*, and *Flavobacteriaceae* species, which encode more peptidases overall and more threonine peptidases, were predominantly linked to aquatic or soil environments (Figure 8A and Supplementary Figure S7 and Supplementary Table S4).

In the *Actinobacteria*, differences among clades were more strongly related to the numbers of peptidase genes than to the types of secreted peptidases (Figure 8B) but were still highly correlated with the environmental microhabitat in which a taxon was found. For example, *Actinobacteria* families associated with animals, such as *Propionibacteriaceae*, *Coriobacteriaceae*, *Bifidobacteriaceae*, and *Corynebacteriaceae*, possessed a lower overall abundance of secreted peptidases, predominantly of the serine, metallo-, and cysteine super-families, compared to aquatic or soil *Actinobacteria*, such as *Streptomycetaceae*, *Pseudonocardiaceae*, *Nocardiaceae*, *Micromonosporaceae*, and *Actinoplanaceae* (Figure 8B and Supplementary Table S5) (*p* < 0.001). Notable exceptions were host-associated *Mycobacteriaceae* genomes that encode significantly more secreted peptidases than other *Actinobacteria* associated with animal environments (Figure 8B and Supplementary Table S5) (*p* < 0.001). By contrast, *Frankiaceae*, which form nitrogen-fixing root nodules in several families of plants, possessed a low abundance of secreted peptidases compared to other aquatic or soil *Actinobacteria* (*p* < 0.001). Principal coordinate analysis of *Actinobacteria* peptidases showed a significant separation among taxonomic families in the relative abundance of secreted peptidases (*p* < 0.001) and their environment (*p* < 0.001) (Supplementary Figure S8).

## Discussion

Serine, metallo-, and cysteine peptidases are the dominant (∼90%) intracellular and extracellular proteolytic enzymes of *Archaea* and *Bacteria*, whereas aspartic and threonine peptidases contribute <10% to the total (Page and Cera, 2008). Intracellular peptidases are often involved with protein turnover and regulatory functions, whereas extracellular or secreted peptidases are typically viewed as an energetic investment of the organisms that is returned via the acquisition of carbon and nitrogen through enzymatic degradation of proteinaceous material in the environment (Chróst, 1991; Geisseler and Horwath, 2008).

Secreted peptidase diversity varied between *Archaea* and *Bacteria*, suggesting the potential for specialized peptidase functions and optimization among taxa. This variation may be related to differences in the catalytic residues of the active site; these biochemical differences may provide specific adaptive advantages to different taxa under varying environmental conditions. As a general hydrolytic mechanism, a nucleophilic amino acid residue or water molecule is activated to attack a peptide carbonyl group, cleaving a peptide bond. In the case of serine, cysteine, and threonine peptidases, the histidine residue of a catalytic triad activates the serine, cysteine, or threonine residue, which then serves as the nucleophile that splits the peptide bond (Rao et al., 1998; Theron and Divol, 2014). Alternatively, for aspartic and metallopeptidases the aspartic acid residue or an enzyme-bound metal cofactor activates a water molecule to act as the nucleophile for the hydrolysis (Wu and Chen, 2011; Theron and Divol, 2014).

Bacterial species generally possess more secreted peptidases per genome and have a more diverse repertoire of secreted peptidase families compared to archaeal species (Figure 2, 3). This may confer greater flexibility on *Bacteria* to generate different types of extracellular proteolytic enzymes in response to specific environmental conditions depending on their demand for carbon and nitrogen, resulting in consistently high levels of overall peptidase activity *in situ*. This also suggests that *Bacteria* could be more competitive in obtaining organic nitrogen from the environment compared to *Archaea*. Empirical studies have implicated *Bacteria* to be the dominant contributor to proteolytic activity in soils (Watanabe and Hayano, 1993, 1996; Katsuji et al., 1994; Watanabe et al., 2003; Sakurai et al., 2007), and bacterial isolates from *Bacillus*, *Pseudomonas*, and *Flavobacterium-Cytophaga* have been shown to be important agents of proteolysis, acting as the main sources of soil peptidase activity (Bach and Munch, 2000; Vranova et al., 2013). Our analysis shows that these genera also have a high richness and abundance of secreted peptidases, consistent with their high soil peptidase activities.

At the super-family level, much of the variability in peptidase profiles between genomes of prokaryotic taxa was linked to differences in counts of less common peptidases, namely aspartic peptidases in *Archaea* and threonine peptidases in *Bacteria* (Figure 1, 3). Differences in the complement of secreted peptidases may reflect their adaptation to environmental conditions, such as temperature or pH. Serine, cysteine, and metallo- peptidases are generally optimized and active at neutral to alkaline pH (Rao et al., 1998; Rawlings, 2016), whereas aspartic peptidases generally exhibit high proteolytic activity in acidic conditions (Rao et al., 1998). Our analyses indicate that these enzymatic pH optima reflect the environments in which they are found. For example, three peptidase families – A05, A37, and S53 – were enriched in archaeal acidophile genomes (Figure 6). All three of these peptidase families have been shown to have optimal endopeptidase activities at low pH (Rawlings et al., 2018). Additionally, peptidases of the S53 family appear to be novel endopeptidases within the serine peptidase super-family. These enzymes encode a catalytic triad consisting of Glu, Asp, and Ser residues, as well as an additional Asp residue in the oxyanion hole of the active site (Wlodawer et al., 2003). This active site arrangement stands in contrast to the traditional Asp, His, Ser triad observed in the more common serine S08 peptidases, and effectively relies on two additional acidic residues for activity. These active site arrangements likely relate to the activities of S08 and S53 peptidases in different pH environments, and may account for the observed strong negative correlation of the presence of S08 peptidases within acidophilic genomes. Therefore, the variation in the diversity of peptidase super-families encoded by microbes appears to be at least partially influenced by optimization of catalytic site to specific environmental conditions.

In *Bacteria*, there is a significant difference between the secreted peptidase composition of Gram-positive and Gram-negative bacteria. These two groups of *Bacteria* differ in cell wall structure (Vollmer et al., 2008; Brown et al., 2015), which may influence their environmental distributions. In Gram-positive bacteria, extracellular enzymes could either be restricted to the cell wall and/or eventually diffuse into the environment (Chróst, 1991). In Gram-negative bacteria, digestive enzymes need to be secreted beyond the outer membrane in order to stimulate the degradation of polymers (Chróst, 1991). The distinction between these enzyme secretion strategies may influence the types of extracellular proteolytic enzymes encoded by these two groups of *Bacteria*. Practically, Gram-positive bacteria may secrete more free extracellular enzymes to the environment in comparison to Gram-negative bacteria with more membrane-bound secreted enzymes (Chróst, 1991; Vollmer et al., 2008; Brown et al., 2015), however, we did not observe a significant difference in the average number of secreted peptidases encoded in the genomes of taxa from these two bacterial groups (*p* = 0.056).

The conservation of secreted peptidase complements between pairs of archaeal and bacterial taxa was found to have a moderate positive correlation with phylogenetic relatedness across the prokaryotic tree of life. For both *Archaea* and *Bacteria* this relationship weakens rapidly as phylogenetic distance increases and, for bacterial taxa at least, the relationship is only significant up to approximately the family-level taxonomic equivalent of phylogenetic similarity. These patterns may be due in some part to horizontal gene transfer of peptidases, which may act to conserve features between more closely related taxa that more commonly exchange genes via this mechanism (Lawrence and Hendrickson, 2003; Choi and Kim, 2007). As discussed below, this conservation may also be partially attributed to a confounding correlation between phylogeny and environmental microhabitat of the taxa considered, given that phylogenetically-related taxa often inhabit grossly similar environments. Thus, our analyses are necessarily limited by the definition of microhabitat used here, which may insufficiently define the true microhabitats of each taxon and the corresponding relationship to functional specialization of peptidase families within those habitats. Despite this potential limitation, these results stand in contrast to the insignificant relationship observed for glycoside hydrolase (GH) profiles and phylogenies of prokaryotes that was defined using a similar approach (Berlemont and Martiny, 2013). Various technical and analytical reasons could account for this discrepency (e.g., differences in databases, classification schemes, and methodological details). However, stronger conservation of peptidase vs. GH content in genomes could also indicate biologically-driven differences in selective pressures on the different enzymatic types, despite their similar general functional roles in modifying cellular components and obtaining resources via secreted degradative enzymes. Further work will be needed to better define the roles of these important enzymes in speciation and competition in the environment.

Most secreted peptidase families encoded in bacterial genomes were determined to have significant phylogenetic signals in their distribution patterns across taxa. These findings agree with previous studies that found conservation of prokaryotic traits that are governed by multiple genes or metabolic pathways (e.g., spore formation, oxygenic photosynthesis; Goberna and Verdú, 2016; Barberán et al., 2017), suggesting that trait conservation and phylogenetic signal strength is not exclusively linked to increased trait complexity. Peptidase families of archaeal genomes did not show the same level of conservation as in *Bacteria*, a result that is likely due to the scant representation of peptidases from individual families in the available archaeal genome dataset, which is a known limitation of this phylogeny-based trait prediction method (Goberna and Verdú, 2016). Non-random distributions were also observed for most super-families when compared to both archaeal and bacterial phylogenies (Supplementary Tables S4, S5). Here, more negative *D*-values, which are indicative of extreme phylogenetic clustering, were typically observed for less common peptidases (e.g., threonine peptidases of *Bacteria* and aspartic acid peptidases of *Archaea*), suggesting specialized adaptive roles for these enzymes based on their distinct catalytic mechanisms.

When the conservation and variation of genome-encoded secreted peptidases was examined within more specific bacterial clades (e.g., *Bacteroidetes* and *Actinobacteria* phyla), it was observed that environmental habitat and microbial lifestyle (i.e., “free-living” vs. “animal-associated”) was an important determinant of peptidase content in genomes (Figure 8). Generally speaking, *Bacteroidetes* taxa commonly associated with aquatic or soil environments, such as *Cytophagaceae*, *Sphinobacteriaceae*, and *Flavobacteriaceae*, encoded more total peptidases compared to animal-associated *Bacteroidetes*, such as *Bacteroides* and *Prevotella*. Additionally, although serine and metallo- peptidases were common to all *Bacteroidetes*, threonine peptidases were present almost exclusively in aquatic/soil-derived *Bacteroidetes* taxa, whereas cysteine peptidases were significantly enriched in animal-associated *Bacteroidetes* taxa. Given the nature of nitrogen limitation in most soil and aquatic environments, the ability to readily break down high molecular weight proteinaceous material into amino acid precursors for cell growth or energy generation would be highly favorable (Chróst, 1991; Geisseler and Horwath, 2008; Kolton et al., 2013). The *Flavobacteriaceae* include taxa with different lifestyles and genome sizes, and which are common inhabitants of terrestrial and marine ecosystems. Their ability to successfully compete in such oligotrophic environments may be dependent on their capacity to quickly degrade proteinaceous material to obtain nitrogen as a supplement to their well-established specialization of using carbohydrates for energy and as a carbon source (Bryson et al., 2017). This may account for the enriched proteolytic enzyme repertoire observed for these taxa, which is comprised of many outer membrane-associated and extracellular peptidases (Kolton et al., 2013; Tully et al., 2014). By contrast, host-associated *Prevotella* species present inside the rumen (Wallace et al., 1997; Griswold et al., 1999) or as periodontal pathogens of humans (Gazi et al., 1997; Mallorquí-Fernández et al., 2008) have fewer secreted peptidases compared to soil/aquatic species in the *Bacteroidetes* phylum.

Similar to these trends, *Actinobacterial* families common to soil and aquatic environments (*Streptomycetaceae*, *Pseudonocardiaceae*, *Nocardiaceae*, *Micromonosporaceae*, and *Actinoplanaceae*) were also found to have a greater diversity and abundance of peptidases encoded in their genomes compared to animal-associated taxa. This observation agrees well with our current understanding of the ecology of *Actinomycetes* and their prodigious role as organic matter decomposers in nutrient-limited environments such as soils and freshwaters (Wink et al., 2017). *Streptomyces* species are abundant in terrestrial ecosystems and are well-known for their ability to use a wide variety of insoluble environmental substrates such as animal, plant, fungal, and microbial biomass by diverse extracellular enzymes, including peptidases (Chater et al., 2010). Interestingly, some secreted peptidases from *Streptomyces*, which are strictly regulated by their own inhibitors, are to cannibalize their own mycelial biomass in order to support aerial growth and sporulation when needed (Chater et al., 2010). With a rich repertoire of keratinases (mostly serine and metallo- peptidases), some *Streptomyces* can degrade keratin, an insoluble structural and highly polymerized protein that is commonly found in the outer covering of many animals (Gupta and Ramnani, 2006; Chater et al., 2010; Lange et al., 2016). In other cases, extracellular peptidases from *Streptomyces* may also play a role as an activating mechanism for other secreted proenzymes, such as nucleases, cellulases, and xylanases (Chater et al., 2010).

An exception within the soil-associated *Actinobacteria* with regard to secreted peptidase content are taxa within the *Frankiaceae* family, which are diazotrophic and can be endosymbionts of actinorhizal plants. Mastronunzio et al. 2009 also noted the lower number of secreted hydrolases, including peptidases, associated with *Frankia* strains compared to soil *Actinobacteria*, and speculated that this may make them less harmful to their plant hosts, thereby facilitating nodulation.

This pattern does not hold for rhizobia, however, which is a group of diazotrophic *Alphaproteobacteria* that form root nodules in legumes and that encode a much richer collection of secreted peptidases compared to *Frankiaceae* or other non-N_2_-fixing *Alphaproteobacteria*. Interestingly, rhizobia are known to fix nitrogen only when in a symbiosis with plants because they lack an endogenous oxygen protection mechanism for the nitrogenase enzyme that catalyzes the nitrogen fixation (Pawlowski and Bisseling, 1996). Thus, possessing an abundant collection of extracellular peptidases might be a strategy for free-living rhizobia to scavenge organic nitrogen and carbon from proteins. In contrast, *Frankia* species maintain their ability to fix atmospheric nitrogen to meet their nitrogen demand when free-living, potentially obviating their need to secrete peptidases to scavenge organic nitrogen from the environment (Pawlowski and Bisseling, 1996; Norman and Friesen, 2017).

Most animal-associated *Actinobacteria* were found to have a lower abundance of secreted peptidases in comparison with those taxa associated with aquatic or soil environments. Examples of the former are taxa from the *Bifidobacteriaceae*, which are often found as members of the human intestinal microbiota, especially in unweaned infant guts (Lee and O’Sullivan, 2010). In this environment, proteolytic activity predominantly arises from peptidases in human breast milk (e.g., anionic trypsin, anionic elastase, and plasmin) and from the gastric proteases (e.g., pepsin) (Heyndrickx, 1963; Dallas et al., 2012). Our analysis (Figure 8) showed that *Bifidobacteriaceae* taxa have a limited potential to break down proteins, which might reflect the high abundance of host-derived peptidases that generate bioavailable nitrogen within the gut. Conversely, another group of animal-associated *Actinobacteria*, the *Mycobacteriaceae*, possess large numbers of secreted peptidase genes in their genomes. Most *Mycobacterium* species are pathogenic (e.g., *M. tuberculosis, M. leprae*) (Gagneux, 2018) and their secreted peptidases appear to function in roles other than nutrient acquisition. For example, S01 peptidases, such as MarP or Rv3671c, protect *Mycobacterium* species from high acidic and oxidative conditions inside the host, especially when in a dormant state (Ribeiro-Guimarães and Pessolani, 2007; Biswas et al., 2010; Kugadas et al., 2016; Botella et al., 2017). Other peptidases in *Mycobacteria*, such as MycP1 of the S08 family, cleave proteins of the virulent secretion system as part of the infection process (Abdallah et al., 2007; Ribeiro-Guimarães and Pessolani, 2007; Ohol et al., 2010).

Collectively, these examples support the potential role of environmental microhabitat in selecting for peptidase functions, with the general theme that host-associated bacteria tend to encode fewer secreted peptidases than those taxa that are free-living. There are exceptions to this pattern, however, which appear to be linked to specialized traits of the microbes (e.g., pathogenicity, nitrogen fixation).

Our analysis of peptidase diversity has practical implications for microbial ecology studies of protein degradation. First, our analysis of the microbial potential for secreted peptidase production is a foundation for subsequent research applying transcriptomic or proteomic approaches to determine how this potential is realized by the secretion of peptidases under specific environmental conditions. Second, the current oligonucleotide primers designed to amplify peptidases from environmental DNA using PCR focus on specific peptidase families that are encoded by limited microbial taxa. For example, the *npr* primers are able to detect neutral metallopeptidases of the M04 family primarily associated with *Bacillus* species (23^rd^ most abundant peptidase family), primers for *sub* detect the subtilisin-like S08 peptidase family associated with *Bacillus* species (9th in abundance), and *apr* primers can identify only alkaline metallopeptidases of the M10 family from *Pseudomonas fluorescens* (57th in abundance) (Bach and Munch, 2000; Tsuboi et al., 2014). Therefore, there is a need to design primer sets that are more universal or target a larger diversity of microbial secreted peptidases and that focus on the more abundant families of secreted peptidases (e.g., S11, C40, M23) in order to better capture the protein depolymerization process in environmental samples.

## Author Contributions

TN and DM conceived of the presented idea. RM assisted with data extraction from MEROPS database and the computational analysis approach. TN extracted and processed data from other databases and implemented the computational and statistical analyses and took the lead in writing the manuscript. DM and RM supervised the findings of this work. All authors provided critical feedback and helped to shape the research, analysis and manuscript.

## Conflict of Interest Statement

The authors declare that the research was conducted in the absence of any commercial or financial relationships that could be construed as a potential conflict of interest.

## Footnotes

1 http://merops.sanger.ac.uk

2 http://www.cytoscape.org/

3 http://www.arb-silva.de/
