# Supplementary material for: Distributions of Extracellular Peptidases Across Prokaryotic Genomes Reflect Phylogeny and Habitat
Source: Front Microbiol. 2019 Mar 5;10:413. doi: 10.3389/fmicb.2019.00413 (PMC6411800; doi:10.3389/fmicb.2019.00413)
Supplement: Supplementary file 1 [file Data_Sheet_1.PDF]

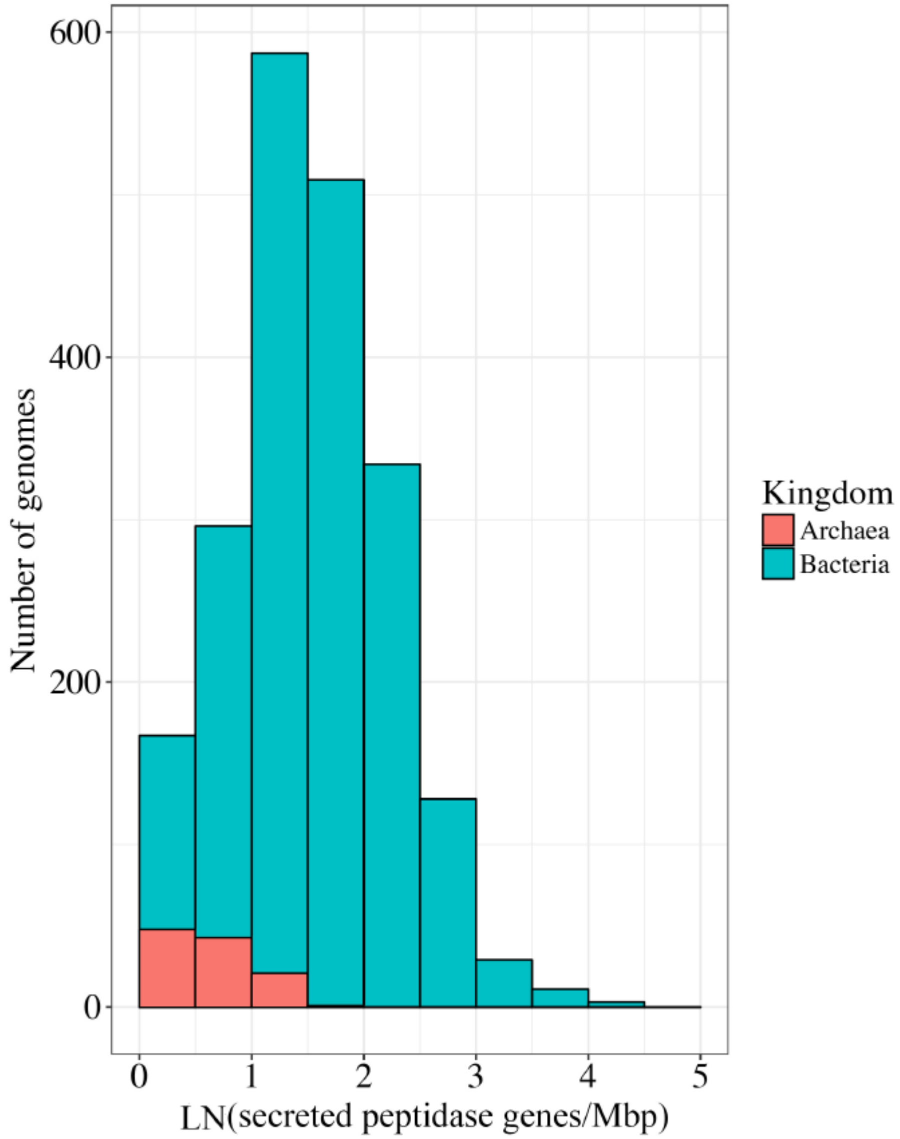

FIG S1 Secreted peptidase gene content of 147 archaeal and 2,191 bacterial genomes (genes/mega base pairs (Mb), natural log transformation). *Archaea* in red and *Bacteria* in blue (Welch two-sample t-test, t=-24.0, p-value < 0.001)

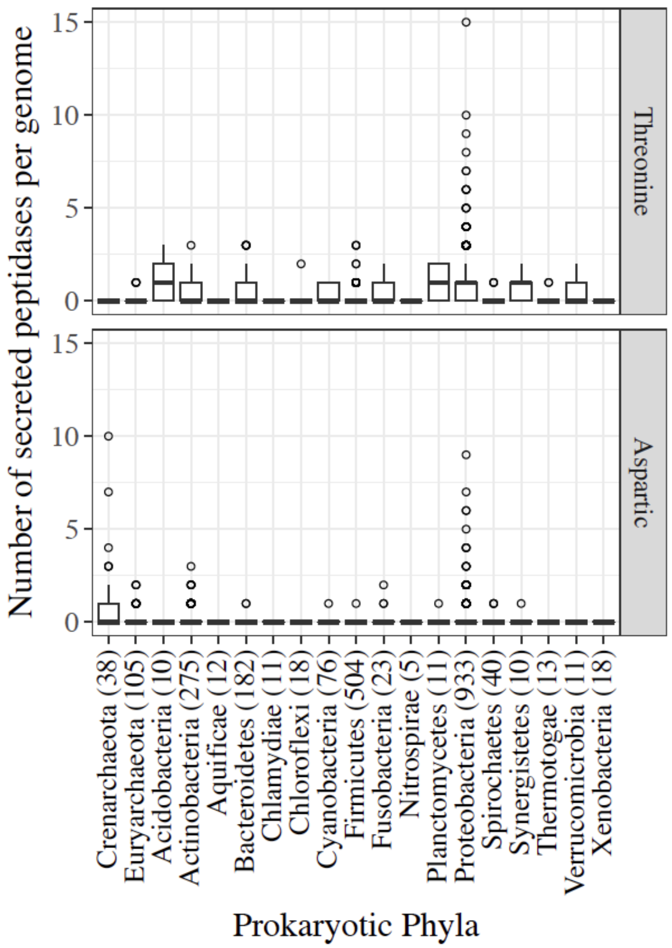

FIG S2 Secreted peptidase gene content (genes/genome) of threonine and aspartic peptidases in prokaryotic phyla. The number of analyzed genomes from each prokaryotic phylum is presented next to the phylum names.

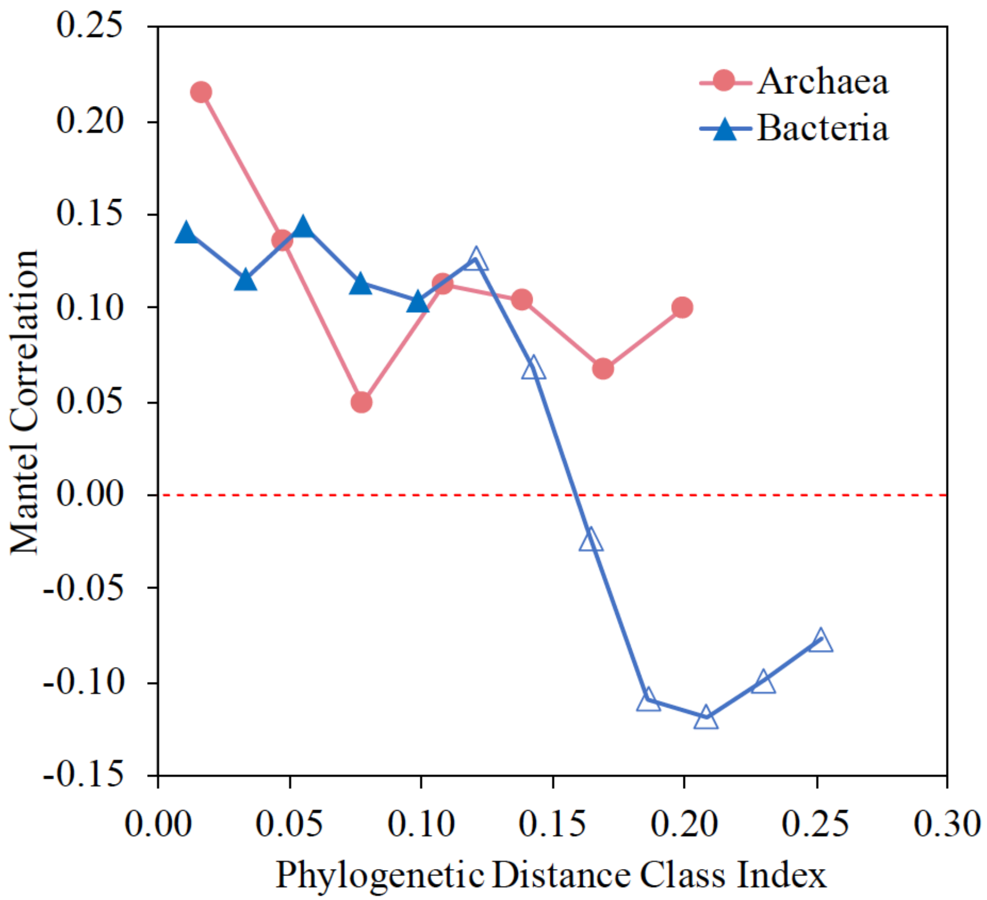

FIG S3 Mantel correlogram between phylogenetic distance and secreted protease profile dissimilarities for archaeal and bacterial taxa based on Pearson's product-moment correlations (p-value < 0.05, filled squares; not significant, open squares).

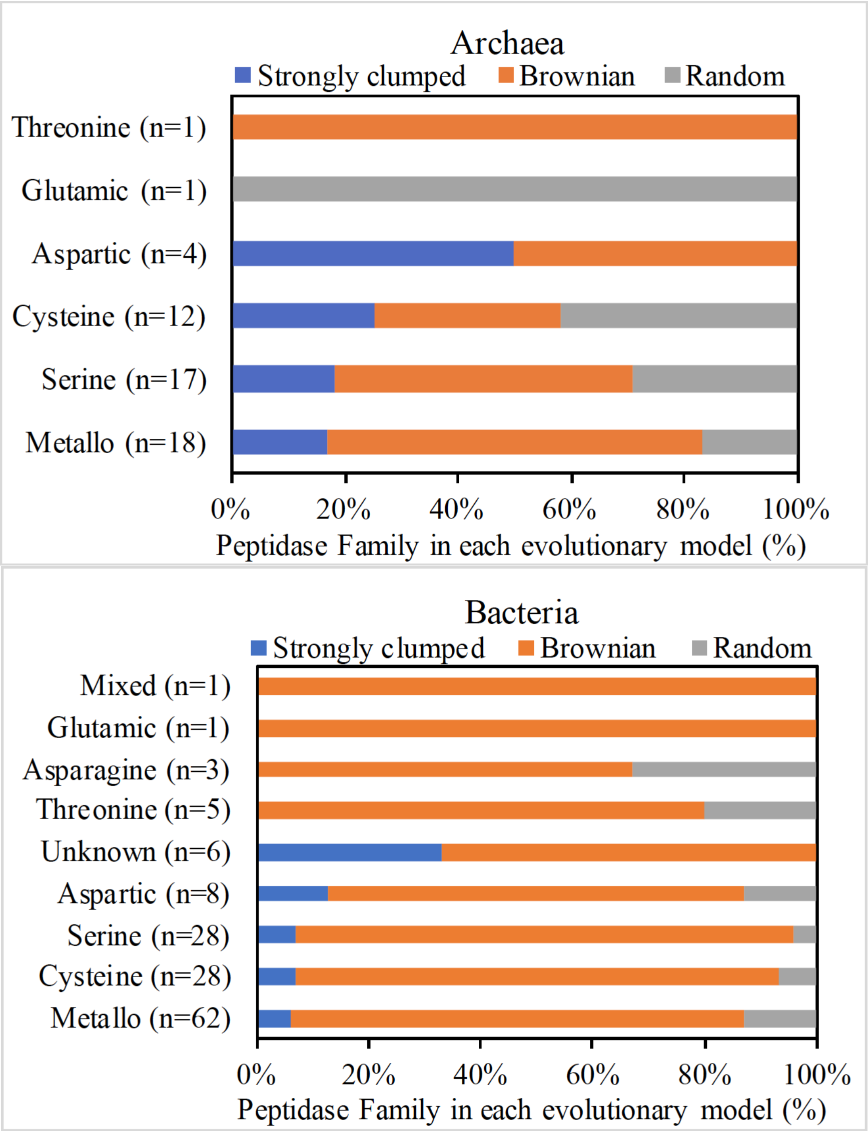

FIG S4 Phylogenetic distributions of secreted peptidase families across archaeal and bacterial taxa.

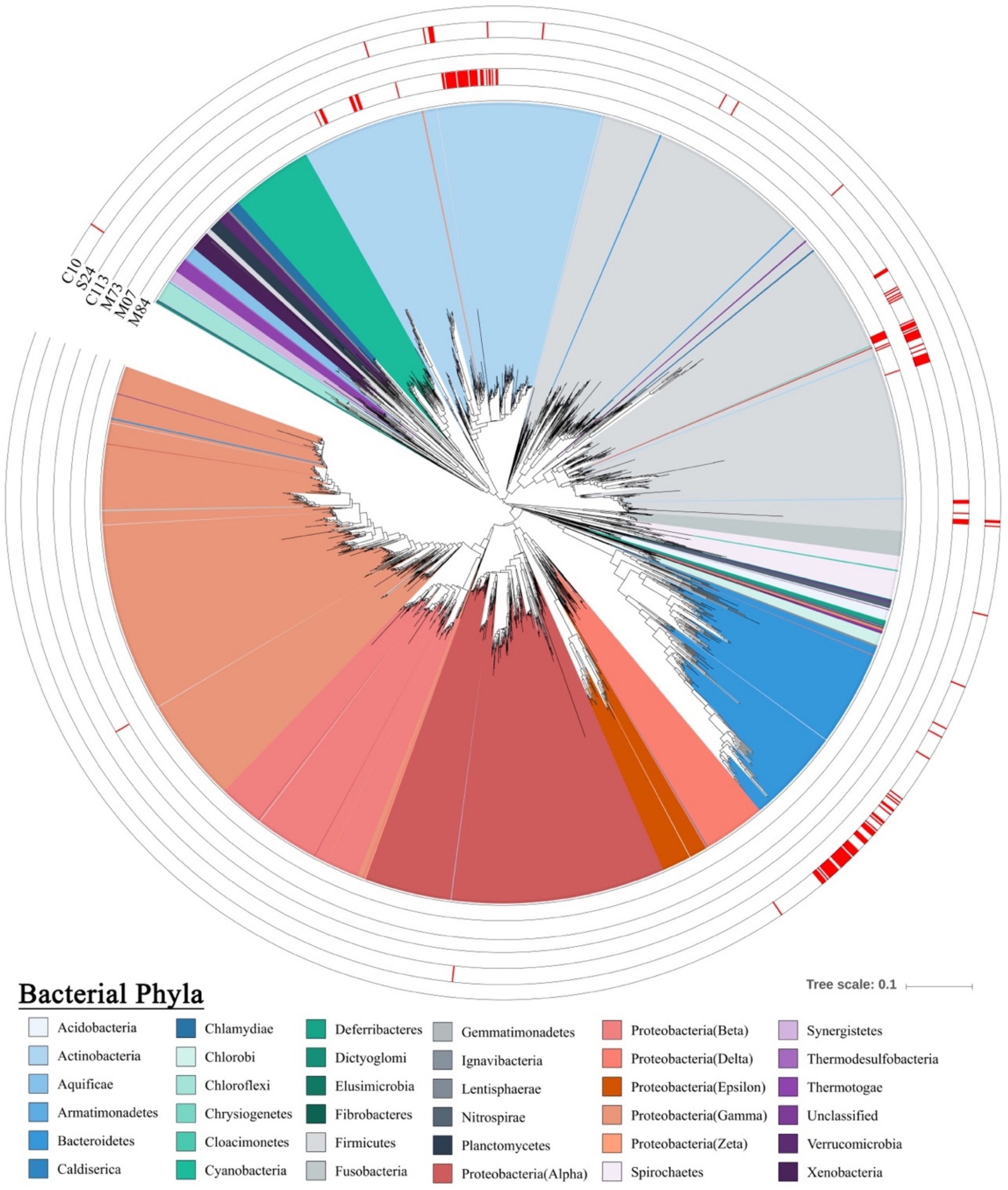

FIG S5 Secreted peptidase families showing clumped distributions within bacterial genomes. Outer tracks show the presence/absence of genes from each secreted peptidase family in each genome (white=absence, red=presence). Inner ring colors with the organism names represented the phylum-level classification of microbial genomes.

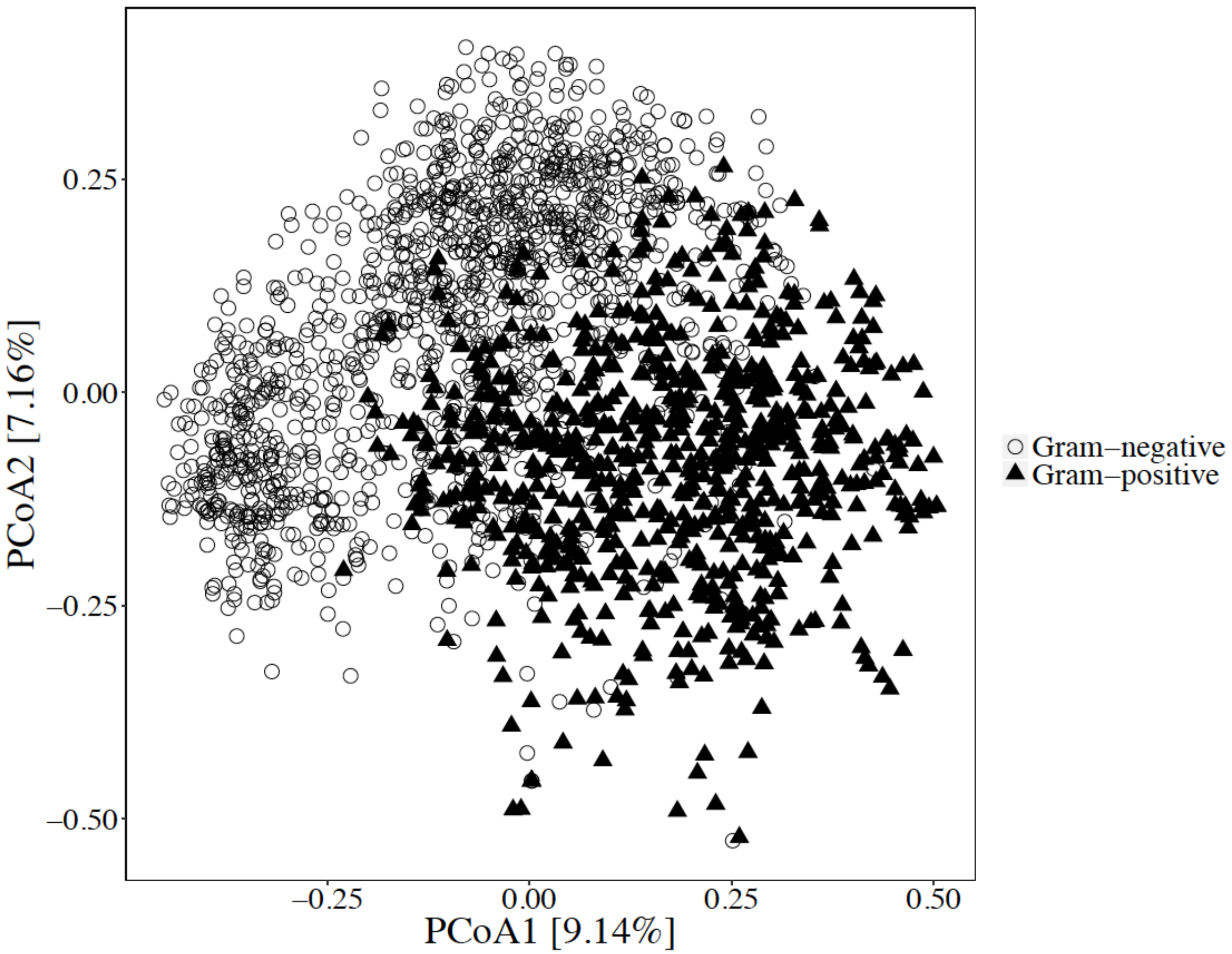

FIG S6 Principal coordinate analysis of prokaryotic genomes based on Bray-Curtis dissimilarities of proportions of secreted peptidase families encoded in each genome. Symbol shapes are coded by either Gram-negative and Gram-positive cell wall classification. Significant differences were observed between Gram-positive and Gram-negative bacteria based on the relative abundance of their secreted peptidase families (p-value < 0.001, F-statistic = 193.3, PERMANOVA).

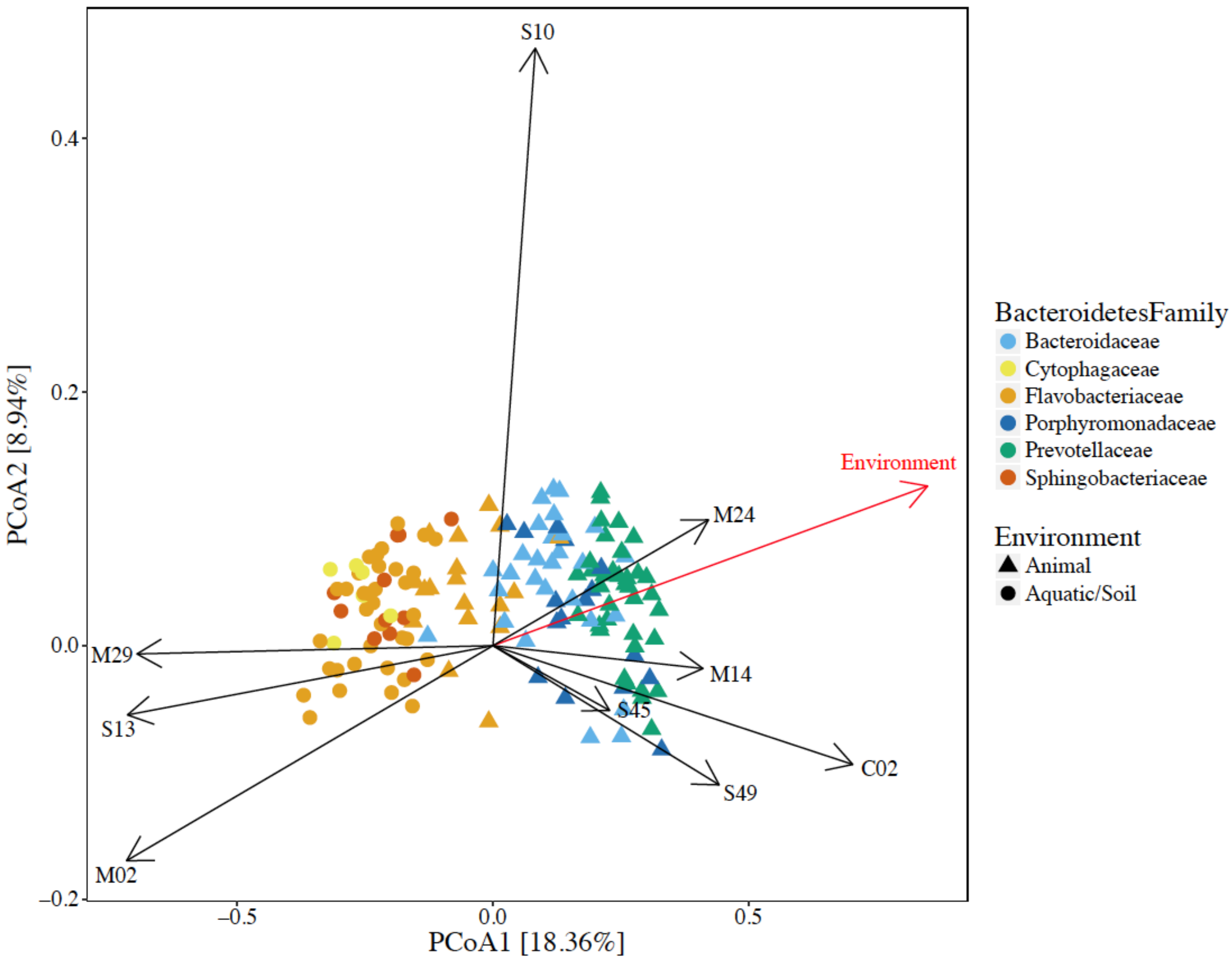

FIG S7 Principal coordinate analysis of prokaryotic genomes based on Bray-Curtis dissimilarities of proportions of secreted peptidase families encoded in *Bacteroidetes* genomes. Symbol shapes are coded by the environment where the microorganisms are commonly found (triangle for animal microbiota and circle for aquatic/soil environment); symbol colors represent different taxonomic families. Vectors lengths are scaled relative to the correlation of individual peptidase families with the two axes shown (Pearson's correlation). Secreted peptidase profiles were strongly correlated with the environment in which each species was associated with (p-value < 0.001, F-statistic = 34.5, PERMANOVA).

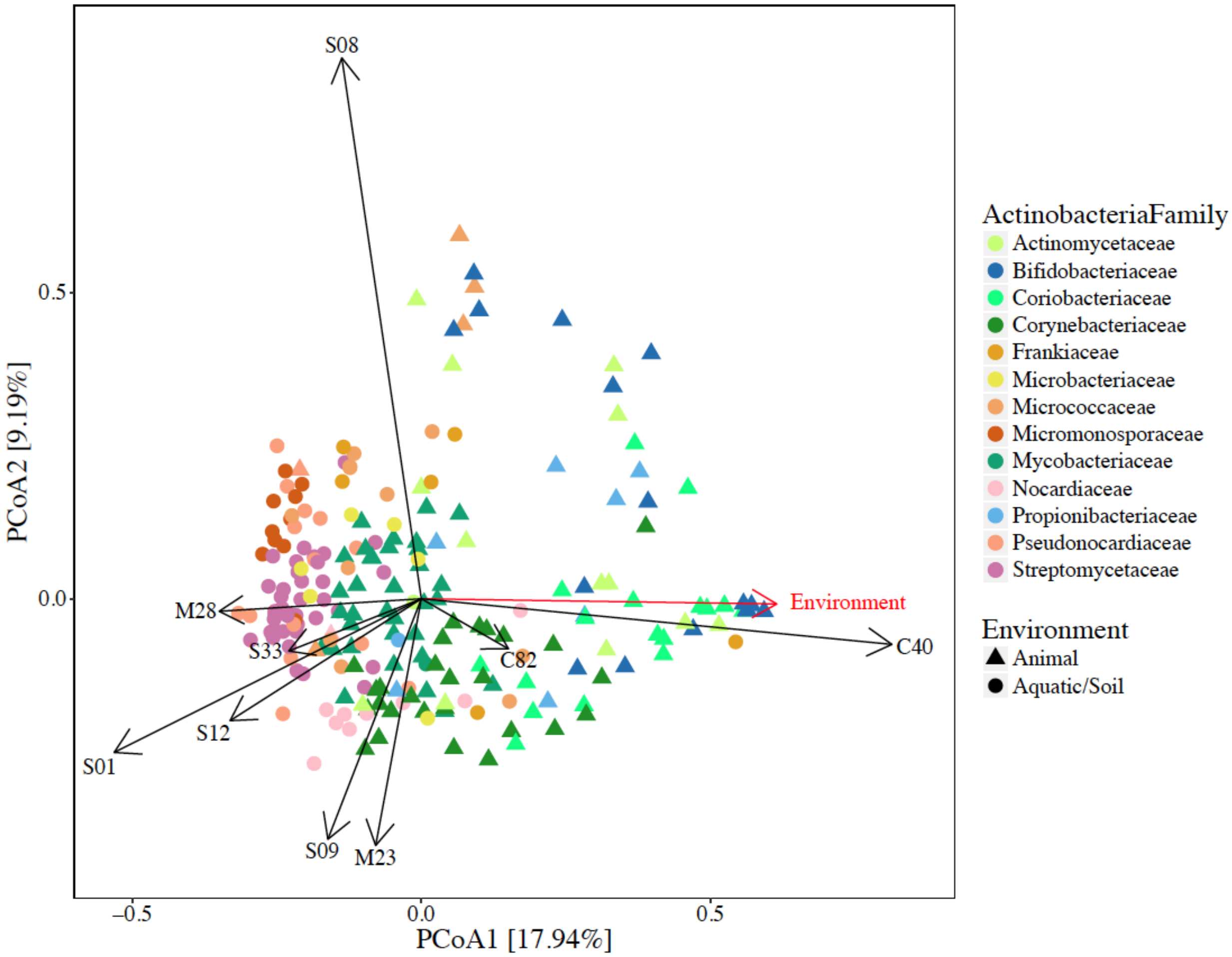

FIG S8 Principal coordinate analysis of prokaryotic genomes based on Bray-Curtis dissimilarities of proportions of secreted peptidase families encoded in *Actinobacteria* genomes. Symbol shapes are coded by the environment where the microorganisms are commonly found (triangle for animal microbiota and circle for aquatic/soil environment); symbol colors represent different taxonomic families. Vectors lengths are scaled relative to the correlation of individual peptidase families with the two axes shown (Pearson's correlation). Ecological niches, on the other hand, also played a strong role in differentiating this function among Actinobacteria (p-value < 0.001, F-statistic = 26.4, PERMANOVA).

TABLE S1 Differences of secreted peptidases among bacterial phyla using Tukey's HSD analysis.

	Acidobacteria	*Actinobacteria*	*Aquificae*	*Bacteroidetes*	*Chlamydiae*	*Chlorobi*	*Chloroflexi*	*Cyanobacteria*	*Firmicutes*	*Fusobacteria*	*Planctomycetes*	*Proteobacteria*	*Spirochaetes*	*Thermotogae*	*Verrucomicrobia*	*Xenobacteria*
Mean number of secreted peptidases	55.3	31.7	3.4	36.3	8.4	7.1	10.4	7.9	17.9	5.7	29.5	28.4	7.4	2.7	20.7	17.1
Acidobacteria			***		*	*	*	***		***			***	***		
Actinobacteria			***	**	*	**	**	***	***	***			***	***		
Aquificae	***	***		***					***		***	***			**	**
Bacteroidetes		**	***		***	***	***	***	***	***		***	***	***		
Chlamydiae	*	*		***												
Chlorobi	*	**		***								*				
Chloroflexi	*	**		***								*				
Cyanobacteria	***	***		***					***		**	***		*		
Firmicutes		***	***	***				***		***		***	***	***		
Fusobacteria	***	***		***					***		***	***			*	**
Planctomycetes			***					**		***			***	***		
Proteobacteria			***	***		*	*	***	***	***			***	***		
Spirochaetes	***	***		***					***		***	***				
Thermotogae	***	***		***				*	***		***	***			***	***
Verrucomicrobia			**							*				***		
Xenobacteria			**							**				***		

p-value: (***) 0-0.001, (**) 0.001-0.01, (*) 0.01-0.05, () > 0.05

TABLE S2. Phylogenetic signal strength of secreted peptidase families across 2,191 bacterial genomes. Significance of clustering is based on the Fritz and Purvis index (D) of each peptidase family trait (presence or absence of genes). Estimated D value defined whether the secreted peptidase distribution would follow “strongly clumped” (D≤0), or “Brownian-like evolutionary” (0<D<1) or “Random” distribution (D≥1).

Peptidase Family	Estimated D	Probability of E(D) resulting from no (random) phylogenetic structure	Probability of E(D) resulting from Brownian phylogenetic structure	Genomes
M07	-0.2259344	0	0.883	47
M73	-0.1694997	0	0.783	32
M84	-0.2318844	0	0.732	10
C113	-0.0912123	0	0.61	11
S24	-0.0186868	0	0.551	9
C10	-0.00685428	0	0.517	59
S46	0.03560422	0	0.401	203
C44	0.1183377	0	0.401	8
M93	0.06470617	0	0.371	51
S28	0.08069442	0	0.316	62
M17	0.09688335	0	0.308	47
M34	0.1920949	0	0.271	16
C15	0.2146679	0	0.262	13
M74	0.08196468	0	0.249	215
C66	0.2367091	0	0.244	12
C02	0.2966722	0	0.244	9
M02	0.108701	0	0.219	86
C25	0.1154511	0	0.213	84
G01	0.3256029	0	0.193	10
U62	0.2398793	0	0.167	26
M88	0.3500109	0	0.161	12
S54	0.3590698	0	0.125	15
M11	0.325439	0	0.115	20
M01	0.09388175	0	0.112	414
S55	0.1978025	0	0.103	59
M19	0.1688879	0	0.078	143
C69	0.1606719	0	0.05	203
M13	0.150051	0	0.048	288
S16	0.2327544	0	0.034	116
N04	0.3161909	0	0.025	55
M86	0.542897	0	0.018	18
S37	0.4474473	0	0.016	24

M28	0.1511733	0	0.015	582
C01	0.1945182	0	0.014	235
A32	0.2915281	0	0.014	85
M35	0.5759979	0	0.014	17
M72	0.4053593	0	0.011	49
M16	0.144704	0	0.009	925
M06	0.2247751	0	0.008	229
M75	0.2263001	0	0.008	210
M66	0.4520076	0	0.008	31
M04	0.2083994	0	0.007	256
S10	0.2441075	0	0.007	180
T02	0.2538085	0	0.007	113
C40	0.1503968	0	0.005	1166
A26	0.3843456	0	0.005	57
S14	0.5553352	0	0.005	23
S06	0.5906663	0	0.004	19
C26	0.6459709	0	0.004	18
M20	0.2435776	0	0.002	277
M49	0.5847863	0	0.002	25
S11	0.2053199	0	0.001	1465
S53	0.3432167	0	0.001	102
M09	0.3744992	0	0.001	88
M64	0.4271057	0	0.001	68
M30	0.4936028	0	0.001	35
A24	0.6858283	0	0.001	24
T03	0.2781851	0	0	772
C82	0.2804334	0	0	910
S13	0.2856551	0	0	649
M03	0.290369	0	0	252
S41	0.3053146	0	0	1163
M14	0.3139296	0	0	335
M38	0.3327629	0	0	304
M96	0.3777077	0	0	192
S12	0.4021022	0	0	1002
S01	0.4097399	0	0	1422
S15	0.4116812	0	0	366
S08	0.4174785	0	0	991
M43	0.4176288	0	0	101
S45	0.4242641	0	0	222

C60	0.4392742	0	0	146
C39	0.4397784	0	0	99
M48	0.4500771	0	0	703
M97	0.4681868	0	0	52
M41	0.4705892	0	0	100
S09	0.4849367	0	0	894
M61	0.493181	0	0	396
C110	0.4960267	0	0	83
M10	0.5026735	0	0	100
C59	0.5149821	0	0	200
S66	0.538241	0	0	84
M15	0.5439112	0	0	543
S49	0.5541239	0	0	269
C14	0.5754452	0	0	110
M26	0.5765666	0	0	41
C93	0.5819775	0	0	73
M24	0.5975717	0	0	74
M36	0.5997786	0	0	64
C13	0.6169344	0	0	29
S33	0.6301359	0	0	426
M23	0.6359832	0	0	1360
M50	0.6435465	0	0	36
S51	0.6527886	0	0	63
C56	0.6592502	0	0	107
A08	0.6680078	0	0	63
C11	0.6738293	0	0	73
C83	0.6841772	0	0	31
M12	0.6982088	0	0	58
U69	0.7769681	0	0	79
S26	0.7917711	0	0	108
M79	0.729761	0.001	0	32
P01	0.7922505	0.003	0	33
C51	0.5217914	0.005	0.05	10
M05	-0.2278856	0.009	0.651	3
T05	0.3934865	0.009	0.216	6
A28	0.7261397	0.009	0	20
M57	0.6577306	0.021	0.015	11
A01	-0.339028	0.029	0.689	2
C47	0.6565088	0.034	0.028	10

M81	0.6174429	0.035	0.065	8
U72	-1.742503	0.052	0.848	1
M60	0.7914669	0.066	0.001	14
U73	0.4583012	0.069	0.233	4
M08	0.7195649	0.116	0.029	7
M18	0.133928	0.128	0.476	2
S73	0.1524141	0.142	0.462	2
M98	0.8741101	0.192	0	13
S59	-0.8006927	0.193	0.672	1
M54	0.7690595	0.243	0.051	5
C19	0.6379069	0.262	0.163	3
U32	-0.04075472	0.316	0.51	1
T07	0.6669495	0.356	0.236	2
N11	0.1935319	0.36	0.428	1
A02	0.1646482	0.371	0.44	1
U74	0.8831385	0.388	0.03	5
M76	0.4561855	0.416	0.392	1
C46	0.7938488	0.47	0.301	1
M56	1.001749	0.511	0	12
N10	1.007016	0.534	0.067	3
M55	1.10351	0.553	0.245	1
S50	1.221841	0.585	0.198	1
M82	1.068431	0.606	0.006	5
M32	1.675042	0.682	0.143	1
M42	1.127865	0.754	0	11
C89	1.484572	0.756	0.028	2
C45	1.096967	0.756	0	15
M44	2.734743	0.819	0.077	1
A11	3.046838	0.874	0.018	1
M78	3.816493	0.915	0.027	1
T01	6.425802	0.982	0	1
M85	3.134598	0.994	0	2

TABLE S3 Phylogenetic signal strength of secreted peptidase families across 147 archaeal genomes. Significance of clustering is based on the Fritz and Purvis index (D) of each peptidase family trait (presence or absence of genes). Estimated D value defined whether the secreted peptidase distribution would follow "strongly clumped" (D≤0), or "Brownian-like evolutionary" (0<D<1) or "random" distribution (D≥1).

Peptidase Family	Estimated D	Probability of E(D) resulting from no (random) phylogenetic structure	Probability of E(D) resulting from Brownian phylogenetic structure	Genomes
C56	-0.8365722	0	0.915	4
A05	-0.3056731	0	0.871	16
S53	-0.293705	0	0.862	18
S16	-0.1277893	0	0.751	44
S12	0.02741457	0	0.447	16
M10	0.04843905	0	0.432	16
M01	0.1706628	0	0.336	8
M79	0.2243264	0	0.179	20
C01	0.3023148	0	0.086	26
S01	0.3590775	0	0.069	20
S49	0.4882171	0	0.004	48
S08	0.4885586	0	0.002	70
M28	0.06883237	0.002	0.478	5
S09	0.545091	0.002	0.012	20
M67	-1.42327	0.004	0.867	2
A37	0.245117	0.005	0.29	6
M48	0.5998375	0.026	0.031	10
C39	0.3796129	0.028	0.241	5
S33	0.4785832	0.028	0.131	7
A22	0.6635692	0.044	0.014	11
A31	-1.788827	0.054	0.911	1
M84	-0.00896638	0.09	0.566	2
M13	0.4058199	0.115	0.317	3
M82	-0.02828574	0.125	0.554	2
C69	0.5660381	0.125	0.185	4
S26	0.7652234	0.125	0.006	11
C110	0.2406773	0.177	0.416	2
T03	0.6611962	0.18	0.145	4
M03	0.3919442	0.244	0.373	2
C19	-0.5283647	0.296	0.558	1
C51	-0.6074144	0.302	0.559	1
S60	-0.4075937	0.321	0.527	1

M43	0.1791917	0.386	0.42	1
S41	0.2983447	0.39	0.381	1
S54	0.7255929	0.406	0.237	2
M30	0.8668889	0.421	0.092	3
M14	0.9940787	0.482	0.002	7
M38	0.6596704	0.496	0.309	1
M09	0.8770507	0.498	0.278	1
S45	1.033513	0.557	0.015	5
S24	1.318157	0.569	0.23	1
M26	1.081546	0.597	0.008	5
S13	1.134787	0.602	0.043	3
C26	1.127768	0.618	0.029	3
M61	1.651654	0.638	0.17	1
C11	1.22681	0.642	0.067	2
M54	1.262134	0.659	0.048	2
G01	2.230843	0.71	0.106	1
C25	2.719368	0.784	0.063	1
C14	1.495708	0.839	0.003	3
C40	3.340966	0.864	0.041	1
S15	1.629052	0.877	0.004	3
S59	3.831459	0.913	0.007	1

TABLE S4 Statistical differences of secreted peptidases between *Bacteroidetes* families using Tukey's HSD analysis.

	Bacteroidaceae	*Cytophagaceae*	*Flavobacteriaceae*	*Porphyromonadaceae*	*Prevotellaceae*	*Sphingobacteriaceae*
Mean. Secreted peptidases	36.91	54.86	33.67	34.94	29.42	54.73
Bacteroidaceae						
Cytophagaceae			*		*	
Flavobacteriaceae		*				**
Porphyromonadaceae						
Prevotellaceae		*				**
Sphingobacteriaceae			**		**	
p-value: (***) 0-0.001, (**) 0.001-0.01, (*) 0.01-0.05, () > 0.05						

TABLE S5 Statistical differences of secreted peptidases between *Actinobacteria* families using Tukey's HSD analysis.

	Actinomycetaceae	*Bifidobacteriaceae*	*Coriobacteriaceae*	*Frankiaceae*	*Microbacteriaceae*	*Micrococcaceae*	*Micromonosporaceae*	*Mycobacteriaceae*	*Nocardiaceae*	*Propionibacteriaceae*	*Pseudonocardiaceae*	*Streptomycetaceae*
Mean. Secreted peptidases	9.42	7.25	7.22	6.67	17.50	17.20	64.70	34.69	43.30	12.13	50.13	72.38
Actinomycetaceae							***	*	**		***	***
Bifidobacteriaceae							***	***	***		***	***
Coriobacteriaceae							***	***	***		***	***
Frankiaceae							***		*		***	***
Microbacteriaceae							***				*	***
Micrococcaceae							***				***	***
Micromonosporaceae	***	***	***	***	***	***		**		***		
Mycobacteriaceae	*	***	***				**					***
Nocardiaceae	**	***	***	*								**
Propionibacteriaceae							***				**	***
Pseudonocardiaceae	***	***	***	***	*	***				**		*
Streptomycetaceae	***	***	***	***	***	***		***	**	***	*	
p-value: (***) 0-0.001, (**) 0.001-0.01, (*) 0.01-0.05, () > 0.05												
